# Activity of zero-valent sulfur in sulfidic natural waters

**DOI:** 10.1186/s12932-014-0013-x

**Published:** 2014-08-19

**Authors:** George R Helz

**Affiliations:** Chemistry and Biochemistry, University of Maryland, College Park, 20742 MD USA

**Keywords:** Polysulfide, Sulfur, Octasulfur, Euxinic basins, Sulfide, Thioanions

## Abstract

**Background:**

Ionic and molecular carriers of dissolved (filter-passing) zero-valent sulfur (S^0^) in anaerobic natural waters include polysulfides, S_n_^2−^, molecular S_8_(aq), organic macromolecules and certain higher valent thioanions. Because S^0^ is rapidly transferred among these various carriers, its biogeochemical roles in such processes as dehalogenation of organic compounds, chelation of trace metals, and anaerobic microbial metabolism are not determined solely by one ionic or molecular species. Here, S^0^ is treated collectively as a virtual thermodynamic component, and computational as well as graphical methods for quantifying its activity (a_S_0) in natural waters are presented. From a_S_0, concentrations of the ionic and molecular carriers of S^0^ can be calculated easily.

**Results:**

Concentration ratios of any two polysulfide ions define a_S_0 (Method I). Unfortunately these concentrations are often too low in nature for accurate quantification with current methods. Measurements of total divalent sulfur (ΣS^-II^), zero-valent sulfur (ΣS^0^) and pH provide a more widely applicable approach (Method II). Systematic errors in ΣS^0^ measurements are the main limit to accuracy of this method at the present time. Alternative methods based on greigite solubility and potentiometry are discussed. A critical comparison of Methods I and II reveals inconsistencies at low ΣS^0^/ΣS^-II^ that imply errors in the thermodynamic data for HS_2_^−^ and S_2_^−^. For samples having low ΣS^0^/ΣS^-II^, an interim remedy is recommended: letting pK_a2_ = 6.3 for all HS_n_^−^ ions.

**Conclusions:**

Newly assembled data for a_S_0 in a selection of anaerobic natural waters indicate that S^0^ is always metastable in the surveyed samples with respect to disproportionation to sulfide and sulfate. In all the surveyed environments, sulfur-rich minerals, such as greigite, covellite and orpiment, are stable in preference to their sulfur-poor cohorts, mackinawite, chalcocite and realgar. The a_S_0 values in the dataset span conditions favoring Hg-polysulfide complexes vs. Hg-sulfide complexes, implying that a_S_0 could affect Hg-methylation rates in nature. No support is found for the common assumption that a_S_0 = 1 in reducing natural waters. This paper calls attention to an urgent need for improved measurement methods, especially for total zero-valent sulfur, as well as new determinations of ionization constants for all HS_n_^−^ species.

**Electronic supplementary material:**

The online version of this article (doi:10.1186/s12932-014-0013-x) contains supplementary material, which is available to authorized users.

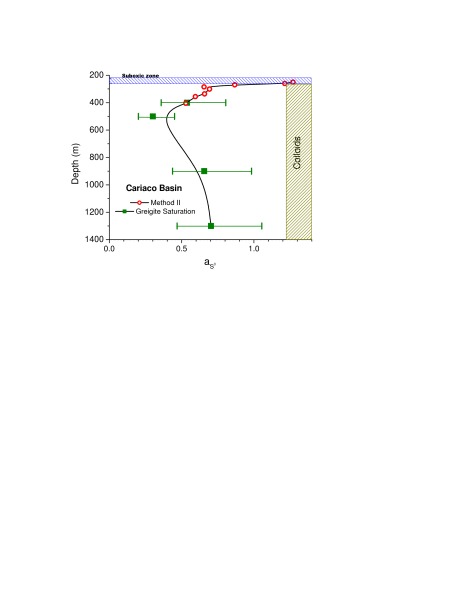

## Background

In aquatic and sedimentary environments, sulfur cycles in a series of steps between the +VI (SO_4_^2−^) and –II (H_2_S/HS^−^) oxidation states. The reductive part of this cycle (+VI →-II) requires biological mediation at earth-surface conditions and is believed to be accomplished intracellularly with little extracellular leakage of the intermediates in most cases [1]. In contrast, the oxidative part can occur via multiple abiotic and biotic pathways that progress through various dissolved intermediates [2]–[4]. The most commonly reported intermediates are zero-valent sulfur, thiosulfate (S_2_O_3_^2−^), and sulfite (SO_3_^2−^) [5]–[8]. In aqueous solutions, dissolved molecular zero-valent sulfur occurs almost entirely as S_8_[9]. If (and only if) free sulfide is present, dissolved zero-valent sulfur will be found also in polysulfides, H_x_S_n_^x-2^ (x = 0 to 2, n = 2 to >8). In principle, zero-valent S can occur in polythionates (O_3_SS_n_SO_3_^2−^, n ≥ 0) and in organic polysulfides or polysulfanes (RS_n_^−^, RS_n_R’, n ≥ 2), but in most sulfidic natural waters these contribute negligibly to total dissolved zero-valent sulfur (ΣS^0^) [6],[10].

Inorganic polysulfides have important functions in nature. By rapidly accepting or donating S atoms, they buffer the activity of zero-valent sulfur (a_S_0):

1 $${S_{n}}^{2-}↔\;{S_{n-1}}^{2-}+\;S^{0}\;K=\frac{a_{S_{n-1}^{2-}}}{a_{S_{n}^{2-}}}\left(a_{S^{0}}\right)$$

Here, S^0^ designates a virtual thermodynamic component, analogous to e^−^, which is the conceptual basis of the well-known pE scale. The standard state for the S^0^ component is chosen so that a_S_0 = 1 at any temperature and 10^5^ Pa pressure in a system at equilibrium with rhombic sulfur. Then, a_S_0 > 1 implies supersaturation and a_S_0 < 1 implies undersaturation of the system with rhombic sulfur. The value of a_S_0 is not linked to the concentration of a specific ion or molecule in solution. Instead, it is a continuous thermodynamic property that is controlled collectively by the assemblage of ions and molecules that participate in rapid, reversible S-atom transfer reactions. Consistent with much qualitative earlier evidence [11], Kamyshny et al. [12] demonstrated that S-atom transfer among polysulfide ions occurs with a characteristic equilibration time of ~10 s. Consequently, polysulfide species can never stray far from equilibrium with one another in the laboratory or the field, and the value of a_S_0 will always be physically well-defined (buffered) in solutions that contain polysulfides as the dominant S^0^ carriers. In this respect, a_S_0 differs from a_e−_ (i.e. 10^–pE^), which can be poorly defined in both in the laboratory and the field because e^−^**-**transfer reactions are often sluggish, and various redox couples in natural waters can be far from equilibrium with one another [13],[14]. As discussed in more detail in Section A of the Additional file 1, buffering of a_S_0 in an aqueous phase by rapid equilibration among polysulfides does not imply or require that polysulfides equilibrate rapidly with particulate phases of sulfur; indeed, owing to slow heterogeneous equilibration rates, the value of a_S_0 in the aqueous phase of a rhombic sulfur suspension is not necessarily unity.

This paper concerns methods for quantifying a_S_0 in natural waters. No tool exists for measuring a_S_0 directly (e.g. a tool analogous to the glass electrode for measuring a_H+_), so a_S_0 must be quantified through thermodynamic modeling. Although experimentalists often employ a_S_0 to describe S^0^-dependent processes in the laboratory, a_S_0 rarely has been determined in sulfidic natural waters. (Among experimentalists in such fields as mineralogy, economic geology and hydrothermal geochemistry, the fugacity of S_2_(g) (f_S2_) has been widely used as an alternate measure of a_S_0 [15]; at 298°K the relationship between these variables is a_S_0 = 10^6.95^f_S2_^½^.)

Knowledge of a_S_0 in natural waters is important primarily for two reasons. First, a_S_0 itself influences the behavior of other elements in nature and constrains the metabolic energy available from microbial oxidation, disproportionation or reduction of zero-valent sulfur. The electric potential available from seafloor fuel cells also depends among other things on a_S_0 in pore waters [16].

In mineral pairs whose compositions differ only in S content (e.g. chalcocite (Cu_2_S) – covellite (CuS), realgar (AsS) – orpiment (As_2_S_3_) or mackinawite (FeS) – greigite (Fe_3_S_4_)), a_S_0 determines which mineral is thermodynamically favored. Even among minerals with nominally the same composition, such as cinnabar – metacinnabar (both Hg_1-x_S), small differences in non-stoichiometry make a_S_0 a possible determinant of which phase is expected to form and persist in nature [17],[18]. Among thioanions of As, Sb and Mo (and probably others), the ratios of oxidized to reduced forms are regulated in sulfidic solutions by a_S_0 as well as a_H_ + [19]–[22]. For example:

2 $$H_{2}{\mathrm{As}}^{\mathrm{III}}S_{3^{-}}+\;S^{0}↔\;{\mathrm{As}}^{V}{S_{4}}^{3-}+\;2H^{+}\;K\frac{a_{S^{0}}}{a_{H^{+}}^{2}}=\frac{a_{\mathrm{As}S_{4}^{3-}}}{a_{H_{2}\mathrm{As}S_{3}^{-}}}$$

Because of reactions of this type, higher valent thioanions of As and Sb, just like polysulfides, must be regarded as carriers of S^0^ in solution. Notice that addition of one S^0^ atom to the non-bonding electron pair in As(III) accomplishes the equivalent of a 2e^−^ oxidation, making S^0^-atom transfer an alternate redox mechanism to e^−^ transfer.

The second principal reason why knowledge of a_S_0 in natural waters is needed is that along with pH and a_HS_–, it can be used to predict polysulfide chain length distributions. An example of chain length’s importance is its effect on trace metal chelation by polysulfides; only S_n_^2−^ ions having n ≥ 4 are able to form chelate rings. In sulfidic waters, chelation by polysulfides is likely to influence strongly the mobility, bioavailability and toxicity of Class b, or soft metal cations (Cu^+^, Ag^+^, Au^+^ and Hg^2+^) [18],[23]–[28]. Another example involves polysulfide-dependent kinetic processes. Explaining the reactivity of polysulfides requires accounting for variations in chain length in response to solution composition [29]. Polysulfide reactivity can control lifetimes of many xenobiotic organic compounds in anoxic environments [30]–[37].

Previously, a convenient graphical approach for determining a_S_0 in laboratory and field samples was presented [38], but this graph is out of date because of major revisions of the thermodynamic data [39]–[41]. The goals of this paper are threefold: 1) to review computational methods and to update graphs for estimating a_S_0, 2) to survey current information about a_S_0 in modern sulfidic water bodies and 3) to explore briefly what this information might imply about biogeochemical processes.

### Quantifying a_S_0

*Method I. Estimating a*_*S*_*0 from measurements of two polysulfide ion concentrations.* During the last decade, development of a fast derivatization method has enabled for the first time measurement of individual inorganic polysulfide ion concentrations in natural waters [42]. When pairs of such ions are abundant enough to be measured, a_S_0 is accessible by a very simple calculation provided pH is high enough that protonation of S_n_^2−^ ions can be ignored. If the formation of a polysulfide ion is represented as follows,

3 $${\mathrm{HS}}^{-}\;+\;\left(n-1\right)S^{0}↔\;H^{+}+\;{S_{n}}^{2-}\;K_{n}=\;\frac{10^{-\mathrm{pH}}a_{S_{n}^{2-}}}{a_{HS^{-}}{\left(a_{S^{0}}\right)}^{\left(n-1\right)}}$$

then for two polysulfide ions that differ in composition by Δn(S^0^) atoms, i.e. S_n_^2−^ and S_n+Δn_^2−^:

4 $$\frac{\left[S_{n+\mathrm{\Delta n}}^{2-}\right]}{\left[S_{n}^{2-}\right]}\approx \frac{a_{S_{n+\mathrm{\Delta n}}^{2-}}}{a_{S_{n}^{2-}}}=\;\frac{K_{n+\mathrm{\Delta n}}}{K_{n}}{\left(a_{S^{0}}\right)}^{\mathrm{\Delta n}}$$

**Figure 1:**
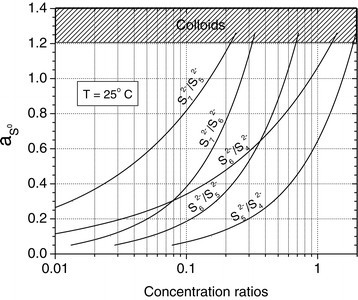
**Relationship of a**_**S**_**0 to ratios of polysulfide ion concentrations.** The hatched region demarks a_S_0 values that are unattainable, except transiently, because rapid precipitation of inorganic colloids occurs. The curves are independent of pH provided pH is high enough that polysulfide ions are not appreciably protonated.

Where [x] and a_x_ respectively represent the molar concentration and activity of species x. Based on this equation, Figure 1 graphically presents relationships between a_S_0 and ratios of those polysulfide ions that are usually most abundant and thus most easily measured.

If a_S_0 indicated in Figure 1 falls inside the hatched area (a_S_0 above ≈ 1.22), then the sample is supersaturated with respect to colloidal sulfur, which will precipitate at least initially as nanoscale, supercooled droplets of liquid sulfur [43],[44]. The value of 1.22 is based on an experimental determination of the solubility of colloidal sulfur [27]. Older experiments that defined the kinetic threshold for rapid colloid precipitation suggest a similar value [43]. The true position of the colloid boundary in natural waters is somewhat uncertain owing to the possibility that natural organic macromolecules could affect its position. Additionally, the temperature and colloid-size dependences of this boundary are not known. It should be emphasized that a_S_0 < 1.22 does not prove absence of colloids. Colloids have been observed in highly dynamic systems when a_S_0 < 1.0 [45]. Possibly these colloids were produced by bacterial sulfide oxidation, which generates colloidal sized, chemically stabilized S^0^-containing substances that are less soluble than abiotic colloids [10],[46],[47].

This approach to quantifying a_S_0 is attractive because activity coefficients of the polysulfide ions reasonably can be assumed to cancel, as done in equation 4, making ionic strength irrelevant. Furthermore, the K_n+Δn_/K_n_ ratio changes slowly with temperature, so in many cases temperature also can be neglected. When a_S_0 has been determined from equation 4, back substitution into equation 3 yields a_HS-_, and these two quantities in turn enable calculation of all polysulfide ion concentrations as well as the concentration of S_8_(aq) in a sample of known pH, ionic strength and temperature. On the other hand, a weakness of this method is its sensitivity to analytical error, as discussed later. In practice, this approach has enjoyed only limited application, because in many samples individual polysulfide concentrations are too small for direct quantification.

*Method II. Estimating a*_*S*_*0 from measurements of pH,* Σ*S*^*0*^*and ΣS*^*-II*^*.* A more widely applicable approach employs thermodynamic modeling based on measurements of pH, total dissolved inorganic sulfide (ΣS^-II^) and total dissolved zero-valent sulfur (ΣS^0^). Computational methods for implementation of this method have been described previously [10],[27],[38]. A description of the approach is given in the Additional file 1, Section B. If S_8_ and all polysulfides are in equilibrium with one another owing to rapid S-atom transfer reactions, then equation 5 expresses the relationship between dissolved ΣS^0^, a_HS-_, and pH.

5 $$\Sigma S^{0}=8\left[\frac{{\left(a_{S^{0}}\right)}^{8}K_{0}}{\gamma_{0}}\left(1+K_{p}\left(\mathrm{DOC}\right)\right)\right]+\left(a_{HS^{-}}\right)\sum_{n=2}^{8}\left[\frac{\left(n-1\right)\left(K_{n}a_{S^{0}}^{\left(n-1\right)}\right)}{10^{-\mathrm{pH}}}\left(\frac{1}{\gamma_{2}}+\frac{10^{-\mathrm{pH}}}{K_{\mathrm{na}2}\gamma_{1}}+\frac{10^{-2\mathrm{pH}}}{K_{\mathrm{na}1}K_{\mathrm{na}2}\gamma_{0}}\right)\right]$$

Here, *γ*_*q*_ represents the ionic activity coefficient of a species having charge *± q*); γ_o_, represents the activity coefficient of an uncharged species such as dissolved molecular S_8_ or H_2_S_n_, and the various stability constants are defined in Section C of the Additional file 1.

The first term in brackets in equation 5 accounts for dissolved (filter passing) S_8_ both in true solution and physically sorbed to filter-passing organic macromolecules (DOC). Owing to its hydrophobic character, S_8_ is expected to associate physically with dissolved organic macromolecules just as hydrophobic pesticides and similar compounds do [10],[48]. The second term is not meant to account for covalently bound organic S, which usually is excluded from dissolved ΣS^0^ by the analytical procedure. Probably, the second term oversimplifies the mechanisms by which DOC can affect S^0^ behavior, but it represents a reasonable description given the current level of knowledge. Synthetic surfactants at concentrations above their critical micelle concentration (CMC) are known to solubilize S^0^ in the same way that they solubilize fats [49]. This mechanism is neglected in Equation 5 because current estimates of the CMC for natural organic matter [50] suggest that DOC rarely achieves sufficient concentrations to form micelles.

Evaluation of a_S_0 for a natural water sample involves finding the root of equation 5, which is an eighth order polynomial, given the temperature, ionic strength, ΣS^0^, pH, and a_HS-_. Unless ΣS^0^ < < ΣS^-II^, evaluating a_HS-_ requires a correction for S^-II^ contained in polysulfides. This can be obtained from:

6 $$\Sigma S^{-\mathrm{II}}=\left(a_{HS^{-}}\right)\left\{\left(\frac{1}{\gamma_{1}}+\frac{10^{-\mathrm{pH}}}{K_{a1}\gamma_{0}}\right)+\sum_{n=2}^{8}\left[\frac{K_{n}a_{S^{0}}^{\left(n-1\right)}}{10^{-\mathrm{pH}}}\left(\frac{1}{\gamma_{2}}+\frac{10^{-\mathrm{pH}}}{K_{\mathrm{na}2}\gamma_{1}}+\frac{10^{-2\mathrm{pH}}}{K_{\mathrm{na}1}K_{\mathrm{na}2}\gamma_{0}}\right)\right]\right\}$$

Equations 5 and 6 can be solved numerically for a_S_0 using tools such as SOLVER in EXCEL, but the labor of setting the problem up is considerable. Fortunately, it will often be the case, especially in neutral to alkaline sulfidic waters, that the amount of ΣS^0^ contributed by free and sorbed molecular S_8_ is negligible. An upper limit to free [S_8_] is about ≈ 0.13 μM (equivalent to ~1 μM as S^0^) because of rapid colloidal S^0^ precipitation above that level [10],[27],[43]. If the terms accounting for free and sorbed S_8_ in equation 6 can be neglected, then dividing equation 5 by equation 6 yields an expression that is independent of a_HS–_:

7 $$\frac{\Sigma S^{0}}{\Sigma S^{-\mathrm{II}}}=\frac{\sum_{n=2}^{8}\left[\frac{\left(n-1\right)\left(K_{n}a_{S^{0}}^{\left(n-1\right)}\right)}{10^{-\mathrm{pH}}}\left(\frac{1}{\gamma_{2}}+\frac{10^{-\mathrm{pH}}}{K_{\mathrm{na}2}\gamma_{1}}+\frac{10^{-2\mathrm{pH}}}{K_{\mathrm{na}2}K_{\mathrm{na}1}\gamma_{0}}\right)\right]}{\left\{\left(\frac{1}{\gamma_{1}}+\frac{10^{-\mathrm{pH}}}{K_{a1}\gamma_{0}}\right)+\sum_{n=2}^{8}\left[\frac{K_{n}a_{S^{0}}^{\left(n-1\right)}}{10^{-\mathrm{pH}}}\left(\frac{1}{\gamma_{2}}+\frac{10^{-\mathrm{pH}}}{K_{\mathrm{na}2}\gamma_{1}}+\frac{10^{-2\mathrm{pH}}}{K_{\mathrm{na}2}K_{\mathrm{na}1}\gamma_{0}}\right)\right]\right\}}$$

**Figure 2:**
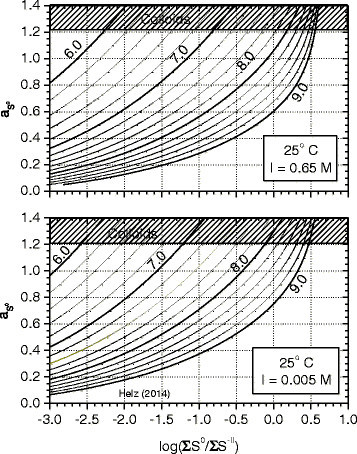
**Graphs for estimating a**_**S**_**0 at 25°C; ionic strengths of 0.65 and 0.005.** Curves are constant pH contours covering pH 6.0 to 9.0 at 0.2 pH unit intervals.

**Figure 3:**
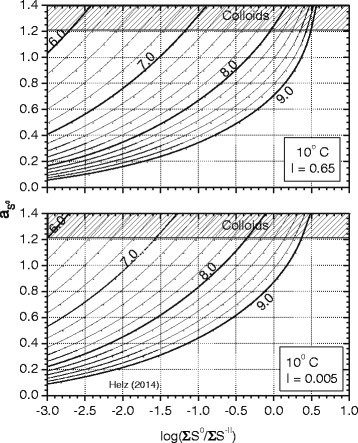
**Graphs for estimating a**_**S**_ **0 at 10°C.**

This equation can be solved for a_S_0 values at a given temperature and ionic strength from only two input quantities, pH and the measured ΣS^0^/ΣS^-II^ ratio. In consequence, 2-D contour plots suffice to present graphical solutions, as done in Figures 2 and 3. (Enlarged versions of these figures are given in Section D of the Additional file 1 for the convenience of readers who might wish to use them to obtain quick approximations of a_S_0 in field or laboratory samples.) Estimates of a_S_0 open the way to computing [S_8_] and the concentrations of all polysulfide species very easily by back substitution into equation 6 and the equilibrium constant expressions. If this procedure reveals that [S_8_] is not negligible relative to measured ΣS^0^, then the approximation inherent in equation 7 is invalid, and a_S_0 must be obtained by a full numerical solution of equations 5 and 6.

Currently, the accuracy of Method II is probably limited chiefly by problems in measuring ΣS^0^. Method II will overestimate a_S_0 if the ΣS^0^ analysis includes contributions from species other than polysulfides and S_8_ in its free and DOC-sorbed states. Colloidal S^0^ is a potential problem in most analytical approaches [7]. Colloids are apt to be more problematic in fresh than saline waters, because hydrophobic particles coagulate rapidly under saline conditions [51]. In fresh as well as saline waters, artifacts due to colloids can be minimized by using dialysis sampling methods [27].

*Critical comparison of Methods I & II.* In a recent study of ground water from wells in Israel, three to four polysulfide ion concentrations were measured directly, and ΣS^0^ was measured by three independent approaches [52]. This rich data set affords an extraordinary opportunity to test Methods I and II against one another and to explore uncertainties. Two wells (Kfar Urya 8 and Tzofar 221) were selected because three independent measurements of ΣS^0^ in their waters were reasonably consistent and because their waters differed greatly in ΣS^0^/ΣS^-II^ ratio. In Section E of the Additional file 1, a_S_0 as well as the concentrations of S_8_(aq) and a number of H_x_S_n_^x-2^ species have been calculated in multiple ways for the two wells using alternate choices of input data.

For Kfar Urya 8 water, in which ΣS^0^/ΣS^-II^ = 0.2, Additional file 1: Table S-2 reveals a level of agreement in the output from the three models based on Method I and the three models based on Method II that seems reasonable in the light of analytical uncertainties. On the other hand, Tzofar 221 waters, which had ΣS^0^/ΣS^-II^ = 0.009, yield wildly discrepant values from seven independent models.

**Figure 4:**
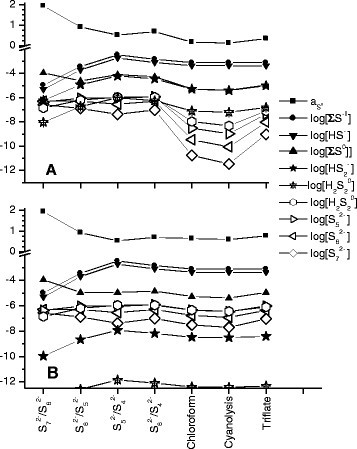
**Comparison of output from four Method I and three Method II models.** Data are from [52] for the Tsofar 221 well (pH = 6.81, I = 0.041, T = 38°C). Labels on the horizontal axis indicate the choices of input data for each of the seven calculations; different pairs of polysulfide species were used in the case of Method I. ΣS^0^ values measured in different ways were used for Method II (the same ΣS^-II^ was used in all Method II models). In the upper graph, thermodynamic data used in the computations were taken from [39]–[41] for S_8_(aq). In the lower graph, pK_na1_ for all H_2_S_n_^0^ species was set to 3.0 and pK_na2_ for all HS_n_^−^ species was set to 6.3. Numerical values on which this figure is based are given in Table S-3.

The problem with the Tzofar 221 data is illustrated in Figure 4A based on the numerical values in Additional file 1: Table S-3A. The desired pattern in Figure 4 would consist of nearly horizontal lines connecting roughly identical concentrations calculated from each of the seven speciation models tested. The actual pattern is quite different. Values of a_S_0 range from 2.04 by Method I using S_7_^2−^ and S_6_^2−^ concentrations as input down to 0.13 by Method II using ΣS^0^ determined by cyanolysis and ΣS^-II^ determined by methylene blue. Because of this large range in a_S_0, ΣS^-II^ calculated by Method I ranges from 3000 to 10 μM, compared to the actual value of 770 ± 38 μM determined by methylene blue. Similarly, ΣS^0^ calculated by Method I ranges from 166 down to 22 μM, compared to actual values from 11 to 4 μM determined by three independent analytical methods. The concentrations of S_4_^2−^, S_5_^2−^, S_6_^2−^ and S_7_^2−^ calculated by Method II using chloroform extraction and cyanolysis data for ΣS^0^ are uniformly below 0.01 μM whereas actual values measured by methyl triflate derivatization are more than 10-fold higher. Clearly, at low ΣS^0^/ΣS^-II^ these thermodynamic models have gone seriously awry; for some variables, the alternate models are inconsistent at the order of magnitude level.

Close inspection of the results reveals two general problems. The first lies in the thermodynamic data. It is apparent from Figure 4A that concentrations of HS_2_^−^ and H_2_S_2_^0^ are much too large and dominate dissolved ΣS^0^ at near-neutral pH when ΣS^0^/ΣS^-II^ is very low. In Method I, where S_4_^2−^ to S_7_^2−^ concentrations are constrained by measurements, the effect of this is to make calculated ΣS^0^ values too large relative to measurements because of the additional S^0^ contained in HS_2_^−^ and H_2_S_2_^0^. In Method II, where ΣS^0^ is constrained by measurements, high HS_2_^−^ and H_2_S_2_^0^ concentrations force a_S_0 downward and make concentrations of S_4_^2−^ to S_7_^2−^ much too small relative to measurements in order to preserve the ΣS^0^ molar balance.

That thermodynamic data for HS_2_^−^ and H_2_S_2_^0^ might be problematic should not be surprising. Current free energies of formation of HS_2_^−^ and H_2_S_2_^0^ rely on a ΔG_f_^0^ value for S_2_^2−^ that has much greater uncertainty than for the other S_n_^2−^ ions [39]. They also rely on pK_a_ values that were not measured, but extrapolated from measurements on S_4_^2−^ and S_5_^2−^[53]. In the extrapolation, pK_a_ values were correlated to reciprocal chain length, n^−1^, where n is the number of S atoms in H_x_S_n_^x-2^ species. This extrapolation method is thrown into doubt by a recent theoretical study which suggests that correlation to n^0^ would be more appropriate [54]. Correlating pK_a_ values to n^0^ rather than n^−1^ lowers the ionization constant of HS_2_^−^ by about four orders of magnitude, greatly diminishing the stabilities of both HS_2_^−^ and H_2_S_2_^0^.

In Figure 4B, pK_a2_ for all HS_n_^−^ species has been set to 6.3, the average of the measurements for S_4_^2−^ and S_5_^2−^. This choice is consistent with the finding in [54] that pK_a2_ values are nearly independent of n, but it is inconsistent with the finding in the same paper that pK_a2_ ≈ 9.4 at all n. Such high pK_a2_ values are rejected here because they contradict experimental evidence [53],[55]. Figure 4B and Additional file 1: Table S-2B show that greatly improved agreement is achieved with this pK_a_ change; only the first two Method I models remain in substantial disagreement.

The second general problem, which explains the remaining disagreement, arises simply from analytical error associated with Method I. In Additional file 1: Table S-4, this is demonstrated by error propagation calculations. It is clear in that table that the precision achieved by Method II is far better than the precision achieved by Method I. However this conclusion must be qualified in two ways. First, Method I precision is impaired in this example because the concentrations of individual polysulfide ions were not far above their detection limits, and thus subject to large relative analytical uncertainty. Method I can be expected to perform better in samples having ΣS^0^ substantially above 10^−5^ M. Second, the excellent *precision* (a measure of *random erro*r) of Method II in this example ignores the above-mentioned *systematic error* in ΣS^0^ determinations. As shown in Additional file 1: Table S-3 (and more extensively in [52]), ΣS^0^ measured by alternate methods varies by nearly 4-fold, giving rise to a similar range in most of the concentrations calculated by Method II. This large range in ΣS^0^ lies outside reasonable estimates of measurement uncertainty, testifying to existence of systematic errors in determinations of ΣS^0^.

Kamyshny and coworkers [45],[52] have used a modified Method I approach to calculate what they call the relative saturation level (RSL), which is identical to a_S_0. They controlled the analytical error problem by a weighted regression method that combines data from several polysulfide ion ratios. For Tzofar 221, their value for RSL (a_S_0) is 0.70 ± 0.12, which agrees within uncertainty with 0.63 ± 0.02 by Method II using ΣS^0^ from chloroform extraction (Additional file 1: Table S-4) or 0.59 ± 0.03 using ΣS^0^ from cyanolysis.

Improvements in measuring ΣS^0^, as well as in pK_a_ data for polysulfides, are urgently needed. However until better measurements become available, assigning pK_na1_ = 3 and pK_na2_ = 6.3 uniformly to all polysulfides seems to be a pragmatic remedy for the disagreement demonstrated in Figure 4. This remedy has been adopted throughout this paper, including in Figures 2 and 3. The entire thermodynamic data set on which calculations in this paper are based is given in Section C of the Additional file 1.

*Possible Other Approaches.* Considering the uncertainties in both Methods I and II, it is prudent to search for additional phenomena that might be used to check a_S_0 values. The a_S_0-sensitivity of greigite solubility offers one possibility. Fe(III)-bearing particles that settle into sulfidic waters release dissolved Fe(II) owing to reductive dissolution. Consequently dissolved Fe(II) concentrations usually increase with water depth until saturation with respect to an Fe-sulfide phase is reached. Below the depth of saturation, Fe(II) usually declines as sulfide continues to rise. Thus in euxinic (sulfidic) waters (i.e. ΣS^-II^ > ΣFe^II^) the horizon where saturation is reached can be recognized by a maximum in the dissolved Fe^II^ concentration profile [56]. (In ferruginous waters, where ΣFe^II^ > ΣS^-II^, sulfide reaches a peak at the saturation horizon instead of Fe.) Mackinawite (tetragonal FeS) is believed to be the first iron sulfide to precipitate in sulfidic waters [57], but if ambient a_S_0 in the saturation zone is high enough, mackinawite can transform to greigite (cubic Fe_3_S_4_).

For example, the Fe^II^ concentration maximum in the Black Sea occurs at 180 m [58]. At this horizon the activity product, Q_FeS_ = a_Fe2+_a_H2S_/10^-2pH^, approaches the solubility product constant for mackinawite and remains nearly constant at greater depth even though dissolved Fe declines [58]. Selective leaching experiments show that the acid-volatile sulfide particles in the deep waters behave analytically as if mostly FeS, with only minor amounts of Fe_3_S_4_[59]. In contrast, the deep waters of Framvaren Fjord and the Cariaco Basin contain mainly Fe_3_S_4_-like acid-volatile sulfide particles, according to selective leaching tests, and contain only minor or negligible FeS [59],[60]. In agreement, aqueous activity products (Q_FeS_) suggest equilibrium with a phase that is distinctly less soluble than mackinawite [60],[61]. These examples imply that even though greigite is a transient in pyrite formation [62], it nevertheless can accumulate and acquire sufficient reactive surface area in some euxinic waters to control dissolved Fe solubility.Greigite’s solubility can be described by reaction 14:

8 $$1/3{\mathrm{Fe}}_{3}S_{4}\left(\mathrm{greig}\right)\;+\;2H^{+}↔\;{\mathrm{Fe}}^{2+}+\;H_{2}S\left(\mathrm{aq}\right)\;+\;1/3\;S^{0}\;K_{\mathrm{greig}}=\frac{a_{Fe^{2+}}a_{H_{2}S}}{10^{-2\mathrm{pH}}}a_{S^{o}}^{1/3}=Q_{\mathrm{FeS}}a_{S^{o}}^{1/3}$$

Therefore in waters equilibrated with greigite,

9 $$a_{S^{0}}={\left(\frac{K_{\mathrm{greig}}}{Q_{\mathrm{FeS}}}\right)}^{3}$$

Unfortunately, the cubic exponent in this equation causes a_S_0 to be very sensitive to analytical uncertainty in Q_FeS_. Additionally, the value of K_greig_ is poorly known. The only previous estimate gave log K_greig_ ≈ 2.6 based on the supposition that a_S_0 = 1.0 in the solutions under study [63]. It has been suggested that this K_greig_ is too large because the material studied may have contained mackinawite impurities [64]. Aside from these problems, not all sulfidic waters come to equilibrium with greigite.

It must be kept in mind that mere presence of greigite does not prove equilibrium. Intracellular greigite is purposefully synthesized and used by magnetotactic bacteria [65], which seem able to do this even when surrounding waters are undersaturated with this mineral. In the Cariaco Basin, sub-nanomolar concentrations of particulate greigite are detected at depths as shallow as 30 m (Supplemental Material for [60]), even though free sulfide is not detected until about 260 m and even though the dissolved Fe^II^ maximum that probably signals true saturation with an Fe sulfide mineral is found at 400 m. At 400 m and below, log Q_FeS_ is reasonably constant, as would be expected in greigite-saturated waters of roughly constant a_S_0.

**Figure 5:**
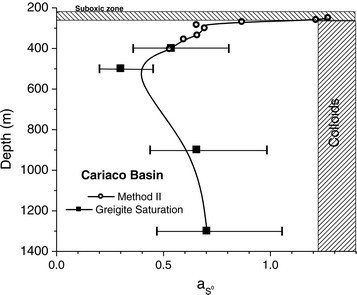
**Activity of zero-valent sulfur vs. depth in the Cariaco Basin.** From 250 to 400 m, a_S_0 was calculated by Method II using data from an April 2007 cruise [66]; salinity = 36.3 and T = 17.5°C. Assuming ±5% uncertainty in ΣS^0^ and ΣS^-II^ measurements and a pH uncertainty of ±0.02, error bars for Method II would fall within the area of the symbols. At 400 m and deeper, a_S_0 was calculated from greigite solubility using data from a May 2008 cruise [60]; error bars depict only measurement uncertainty and are based on an assumption that ΣFe^II^ and ΣS^-II^ are measured to ±5% precision and pH to ±0.02. Log K_greig_ = 2.24 was chosen to make the two methods agree at 400 m.

From published and archived data on the Cariaco Basin [60],[66], a_S_0 can be estimated by Method II from the chemocline at 260 m to 400 m (taking dissolved ΣS^0^ to be the difference between reported total S^0^ and particulate S^0^ values). Unfortunately S^0^ data are not available below 400 m. However, at 400 m and below, where greigite saturation is inferred, a_S_0 can be estimated by equation 15. Figure 5 shows a spliced a_S_0 profile for the Cariaco Basin. To obtain agreement at 400 m, it was necessary to adjust log K_greig_ to 2.24, a reasonable value but slightly lower than Berner’s value of 2.6 [63]. The figure shows that a_S_0 reaches the limit of colloidal S^0^ saturation in the suboxic zone where sulfide oxidation processes are rapid [66]. Below the suboxic zone a_S_0 declines to values on the order of 0.5.

Potentiometry offers another possible approach to checking a_S_0 values. Measurement of Eh (i.e. Pt electrode potential referenced to the standard H_2_ electrode) has fallen out of favor in recent decades because in oxic natural waters irreversible, mixed potentials are obtained, and these are not interpretable with thermodynamics [14],[67]. Nonetheless, early workers demonstrated that reversible potentials are established at inert Pt electrodes in sulfide-polysulfide solutions [68] as well as in sulfidic natural waters [69],[70]. A Nernst equation describing this process can be formulated as follows, where Eh^0^ = −0.0626 V is calculated from thermodynamic tables [71]:

10 $$\mathrm{Eh}\left(\mathrm{volts}\right)=-0.0626+\frac{2.3\mathrm{RT}}{2F}\log\left(\frac{10^{-\mathrm{pH}}}{a_{\mathrm{HS}-}}\right)+\frac{2.3\mathrm{RT}}{2F}\log\left(a_{S^{0}}\right)$$

In more than fifty cores from tidal flats and bogs, Berner [69] measured Eh and E_Ag2S_, the potential of an Ag/Ag_2_S electrode, which can be described by:

11 $$E_{Ag_{2}S}\left(\mathrm{volts}\right)=-0.267+\frac{2.3\mathrm{RT}}{2F}\log\left(\frac{10^{-\mathrm{pH}}}{a_{\mathrm{HS}-}}\right)$$

Combining equations 16 and 17 gives:

12 $$\mathrm{Eh}-E_{Ag_{2}S}=+0.204+\frac{2.3\mathrm{RT}}{2F}\log\left(a_{S^{0}}\right)$$

Thus if a_S_0 = 1.0, then (Eh-E_Ag2S_) would be +0.204. Over a wide range in both Eh and E_Ag2S_, Berner observed (Eh-E_Ag2S_) values falling within a ~0.05 V-wide band that was consistent with a_S_0 values within an order of magnitude of unity; most samples were characterized by a_S_0 < 1.

In experienced hands, Pt electrode potentials can produce a_S_0 values consistent with Method II. For example, the data of Boulègue et al. [72] from the pH 6.9 spring K22 at Puzzichello, in Corsica, yield a_S_0 = 1.57 and a_HS-_ = 0.00063 M by Method II. From equation 16, this is consistent with Eh = −0.161 V, which compares well with the measured Eh of −0.155 ± 0.010 V. The small colloidal S^0^ supersaturation indicated by a_S_0 > 1.22 probably represents a perturbation induced as the water emerged from the subsurface [72].

**Figure 6:**
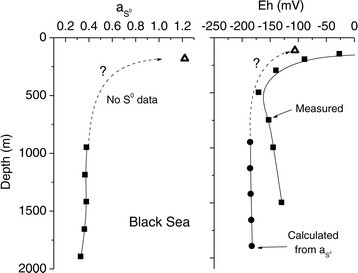
**Black Sea data.** Left: a_S_0 calculated by Method II (data [73],[74]). The total S^0^ data in [73] were assumed to represent dissolved ΣS^0^ because particulate S^0^ at these depths is negligible [75]. Right: Eh measured by Skopintsev (quoted in [76]) compared to values calculated from a_S_0 by equation 15. Open triangles are based on evidence for colloidal S^0^ saturation at the top of the sulfidic zone [77].

However, in other cases Eh calculated from equation 16 is inconsistent with measured Eh for reasons still unknown. The inconsistencies might point to fatal obstacles associated with attempting Eh measurements in sulfidic natural waters. Alternatively, they might simply testify to imperfections in the available data (for example, Boulègue and Michard [70] note that at near-neutral pH it is usually necessary to equilibrate Pt electrodes for several hours to get reproducible readings—something that is hardly ever done in the field). In the lefthand panel of Figure 6, a_S_0 has been estimated by Method II in the Black Sea, and in the righthand panel, Eh values implied by the a_S_0 values are crosschecked against some old Eh measurements. Qualitatively the Eh profiles show similarities, but quantitatively, agreement is poor. Unfortunately, some of this poor agreement might be due to temporal changes in the Black Sea, because the datasets being compared were not obtained contemporaneously.

A key limitation of Eh measurements is their inherent imprecision; a typical measurement uncertainty of ±0.01 V corresponds to a factor of two uncertainty in a_S_0. Nevertheless, Eh is extremely attractive as a possible screening method for a_S_0 in natural waters because it can quite easily be deployed *in situ*, avoiding risks of oxidation, H_2_S(g) loss, S_8_ sorption and pH shifts that can occur when samples must be withdrawn from sulfidic environments for analysis. Additionally, because Eh is in principle a thermodynamic property, it should be unaffected by S^0^ colloids. Because of these attractive characteristics, further research on this approach might be warranted.

## Discussion

*Range of a*_*S*_*0 in Natural Waters.* In addition to the a_S_0 profiles in Figures 5 and 6, a few isolated examples of a_S_0 values are presented in Table 1 in order to illustrate the range observed in nature. These values were all calculated by Method II. The first two entries in the table, Enghien-les-Bains and Puzzichello springs, are found to have quite high a_S_0, probably artifacts due to inclusion of colloidal S^0^ in the ΣS^0^ measurements. Independent evidence for colloids was indeed observed at those sites and attributed to oxidation of sulfide as the deep ground waters mixed with shallow, oxygenated ground waters prior to their emergence in the springs. Lower Mystic Lake deep water as well as pore water from immediately below the sediment-water interface in Lake Croche also have calculated a_S_0 above the colloidal S^0^ limit, hinting at the presence of colloids. Even though the Lake Croche samples were collected by a sophisticated dialysis method that should have excluded colloids exceeding 0.2 μm diameter, their low pH may have promoted post-sampling H_2_S(g) loss through the dialysis membranes (most polymers are extremely permeable to H_2_S gas). Such loss would cause a transient a_S_0 spike and possibly initiate colloid precipitation. Two pore water samples from Great Marsh, a tidal salt marsh subject to intermittent exposure to air, cluster near saturation with respect to colloidal and rhombic S. Although not shown, similar values can be obtained from an independent dataset acquired at this same marsh [78]. On the other hand, pore waters from a Prairie Pothole lake, and bottom waters from Mahoney Lake are appreciably undersaturated with respect to both colloidal and rhombic sulfur and are seen to have a_S_0 in the range of deep euxinic basin waters (Figures 5 and 6).

**Table 1:** **Method II applied to selected natural waters**

Sample	Ref.	ΣS^-II^(mM)	ΣS^0^(mM)	I (mM)	T (°C)	pH	HS^−^(mM)	a_S_0
Enghien-les-Bains, ER2	[79]	1.13	1.65	10	14	7.42	0.53	2.14
Puzzichello K22	[72]	1.64	0.165	21	14.5	6.9	0.633	1.57
Lower Mystic Lake (Ave of deep water)	[32]	7.1	0.33	400	8	6.80	2.30	1.37
Lake Croche +3.5 cm^†^ above sed. surface	[80]	0.0081	0.0010	10*	5*	5.67	0.00019	1.42
Lake Croche −0.5 cm^†^ below sed. surface	[80]	0.011	0.0045	10*	5*	5.96	0.00050	1.71
Lake Croche −3.5 cm^†^ below sed. surface	[80]	0.0037	0.0007	10*	5*	6.05	0.00020	1.36
Great Marsh SU-82, 10–14 cm below sed. surface	[81]	2.15	0.22	1170	15*	7.02	1.08	1.26
Great Marsh SU-82, 50–54 cm below sed. surface	[81]	5.46	0.33	810	15*	7.17	3.26	0.98
Prairie Pot Hole P1 pore water; April	[36]	2.37	0.079	480	15*	8.62	2.29	0.30
Prairie Pot Hole P1 pore water; Sept	[36]	1.95	0.079	480	15*	7.36	1.36	0.77
Mahoney Lake deepest water column, 14 m	[82],[83]	27	0.29	600	14	7.30	18.0	0.58

*When I or T were not reported, estimated values were used. ^†^DOC = 10 mg/L assumed.

In Lake Croche’s low-pH, low-sulfide waters, S_8_ is determined to be the major carrier of ΣS^0^, and in the presence of ≥5 mg/L DOC, most S_8_ will be associated with organic macromolecules. As a result, Croche’s calculated a_S_0 values are sensitive to DOC concentration. At the opposite extreme, because of Mahoney Lake’s much higher pH and sulfide concentrations, S_8_ is negligible relative to polysulfides, and its a_S_0 is consequently insensitive to DOC concentration even though Mahoney’s DOC is extraordinarily high (~800 mg/L [82]).

From the derived a_S_0 and [HS^−^] values in Table 1, the concentrations of S_8_ and all polysulfide ions can be calculated easily from equilibrium constants (values compiled in the Additional file 1). Perhaps surprisingly, the concentrations of polysulfide ions are found not to be simply proportional to a_S_0 or to ΣS^0^. For example, in Lake Croche the calculated concentrations of polysulfides are all <0.002 μM; in contrast, in Mahoney Lake concentrations of S_4_^2−^ and S_5_^2−^ each exceed 20 μM. (The deep waters of Mahoney, and also Lower Mystic, are reported to have a marked yellow color, probably due to polysulfides.) The immense difference implies that a kinetic process dependent on polysulfide concentration, such as degradation of a pesticide [33]–[35], would be >10^4^ faster at the sediment-water interface in Mahoney than in Croche. A rate disparity this large would not be anticipated simply by inspection of either the a_S_0 or ΣS^0^ values in Table 1.

Notice further in Table 1 the non-intuitive relationship between the concentration and the activity of zero-valent sulfur, ΣS^0^ and a_S_0. For example, high values of a_S_0 in Lake Croche are associated with very low ΣS^0^ concentrations, whereas low a_S_0 values in Mahoney Lake are associated with very high ΣS^0^ concentrations; direct proportionality between a_S_0 and ΣS^0^ does not occur. This behavior arises because the analytically measurable quantity, ΣS^0^, is not the concentration of a single chemical species. Rather, it is the concentration of an array of labile species that vary relative to one another depending on solution composition.

Kamyshny and coworkers, using an approach related to Method I, found a_S_0 (which they designated the relative saturation level, RSL) of 0.61 to 0.77 in sulfidic well waters [52]. In Wadden Sea tide pools [45], a_S_0 values were in the range 0.3 to 0.9. Curiously, these latter samples contained visual evidence of sulfur colloids even though a_S_0 < < 1.22. One possible explanation is that the colloids were composed of biogenic sulfur, a hydrophilic substance which is less soluble than both inorganic colloidal sulfur and rhombic sulfur. Biogenic sulfur particles produced by sulfide-oxidizing *Thiobacilli* are characterized by a_S_0 = 0.95 ± 0.02 at 21°C according to data of Kleinjan et al. [46] as analyzed by equations 5 and 6. Probably these microbes enhance their energy harvest from sulfide oxidation by discharging a metabolic S^0^ product, possibly a long-chain polythionate [47], in which a_S_0 is lower than in colloidal or rhombic sulfur.

*Production and Decomposition of S*^*0*^*.* Dissolved zero-valent sulfur is metastable. Given sufficient time, it would spontaneously disproportionate to sulfide and sulfate:

13 $$S^{0}+\;H_{2}O\;\to 0.75H_{2}S\;+\;0.{{25\mathrm{SO}}_{4}}^{2-}+\;0.5H^{+}$$

**Figure 7:**
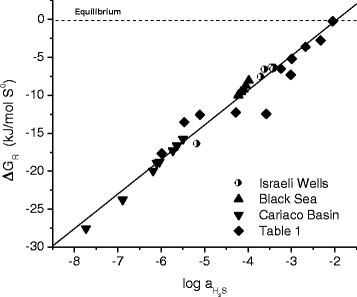
**Free energy available per mole S**^**0**^**by disproportionation of S**^**0**^**to sulfide and sulfate.** Based on data in Figures 5 and 6 and in Table 1 as well as [52]. ΔG_R_ has been plotted against log(a_H2S_) because most of the ΔG_R_ variation in this dataset is due to variation in H_2_S. Zero-valent sulfur is shown to be thermodynamically unstable in all the samples; that is, disproportionation would produce a decrease in free energy. The sample at upper right, lying very close to the equilibrium line, is the extremely sulfidic Mahoney Lake sample. It should be noted that ΔG_R_ values depend on how reaction 19 is written; some previous workers [84] write the reaction to produce one mole of SO_4_^2−^, rather than ¼ mole, and consequently obtain ΔG_R_ values four times larger. This arbitrary choice affects the values on the vertical scale but not the pattern of the data. For reaction 19, ΔG_R_^0^ = +30.124 kJ/mol [71].

Abiotically, this reaction is extremely sluggish under conditions near Earth’s surface (half-life of centuries [29]). However microorganisms are known to catalyze it at high a_S_0, high pH and low a_H2S_, recovering some of the trapped free energy [84],[85]. For the samples discussed in this paper, the available free energy is shown in Figure 7 based on the derived a_S_0 values. Figure 7 shows that disproportionation of S^0^ is uniformly exergonic in these samples, but only slightly so in the extreme case of Mahoney Lake.

Disproportionation is only one path by which S^0^ can be consumed in anoxic environments. Abiotically, S^0^ can be scavenged by pyrite formation [62] and by forming covalently bonded organic sulfur compounds [86]. Owing to their high a_S_0 (Figure 5 and 6), chemoclines manufacture much of the framboidal pyrite found in euxinic basin sediments [62]. Microorganisms also can reduce S^0^ in reactions with hydrogen, methane and other organic compounds [4]. Except near redox interfaces, lack of oxidizing agents curtails oxidative pathways of S^0^ consumption.

In sulfidic waters these loss processes are opposed by production processes mainly involving both biotic and abiotic oxidation of sulfide by various oxidizing agents (Fe^III^, Mn^III,IV^, NO_3_^−^, NO_2_^−^ and of course O_2_) [4],[60]. As Figures 5 and 6 suggest, these production processes are most active near redox interfaces where concentration profiles of oxidizing agents and sulfide overlap to a limited extent. If sulfidic waters occur at depths shallow enough to be in the euphotic zone, anoxygenic photosynthesis becomes a very important source of S^0^. In deeper waters of euxinic basins, sinking particles may be the principal ΣS^0^ source. Detrital Fe^III^-bearing particles that are too refractory to react quickly with sulfide as they fall through the chemocline will be one source [87]. Particles may also carry S^0^, itself. Particle-borne S^0^ would include intracellular S^0^, flocs formed when hydrophobic S^0^ colloids coagulate with detrital and biogenic particles and S^0^ sorbed to such particles. Often, S^0^ from these sources accumulates in elemental form in sediments beneath sulfidic waters [88],[89].

Figure 5 indicates how these opposing processes play out in the Cariaco Basin. In the suboxic zone, owing to very rapid production of S^0^, the calculated a_S_0 reaches the limit imposed by rapid colloid precipitation; S^0^ accumulates in both particulate and dissolved forms [66]. With increasing depth, a_S_0 declines, partly due to much lower production rates in the absence of oxidants and partly due to biological reduction. Sulfur isotope data, which arguably could imply active disproportionation in Cariaco’s deeper waters, are better explained by very slow sulfate reduction rates [90]. Figure 6 suggests that similar processes occur in the Black Sea, but the data are incomplete.

In passing, it should be noted that published polysulfide ion concentrations in the deep Cariaco Basin were calculated in ref [66] on the basis of the common assumption that a_S_0 = 1.0. At 400 m in the Cariaco Basin, the calculated S_5_^2−^ concentration was 0.53 μM under this assumption. Here, where a_S_0 is evaluated by Method II, S_5_^2−^ is calculated to be an order of magnitude less, 0.046 μM. This survey provides no evidence that natural waters tend to be buffered near a_S_0 = 1.0. Enormous errors can be introduced by making this seemingly innocuous assumption, and such errors can mislead assessments of polysulfides’ role in such processes as discussed below.

*Broader Geochemical Implications.* Having for the first time estimates of the range of a_S_^0^ values in a variety of sulfidic waters, it is of interest to consider what these values imply about the geochemistry of elements other than sulfur. Here the effects of a_S_^0^ on trace element speciation and sulfide mineral stability are briefly discussed.

Both sulfide and polysulfide ligands compete for Hg^2+^ in reduced natural waters. At near neutral pH and ΣS^-II^ > 0.1 mM, either HgS_2_^2−^ or Hg(S_n_)_2_^2−^ can predominate, depending on a_S_0 [26]:

14 $$2\left(n-1\right)S^{0}+\;{{\mathrm{HgS}}_{2}}^{2-}↔\;\mathrm{Hg}{{\left(S_{n}\right)}_{2}}^{2‐}\;K\;=\;10^{1.3}$$

The value of n, the number of sulfur atoms in the polysulfide ligands, has not been determined but is likely to be either 4 or 5. From published stability constants [26], the polysulfide complex is predicted to predominate at a_S_^0^ > 0.6 to 0.7 for n = 4 or 5. Thus the a_S_0 boundary separating predominance of the sulfide from the polysulfide complexes at neutral pH falls in the range of a_S_0 values found in natural waters. The Hg(S_n_)_2_^2−^/HgS_2_^2−^ ratio will increase steeply in proportion to a_S_0. Microbial Hg methylation, which is the entry point of Hg into aquatic food chains, is known to be sensitive to Hg speciation, with the polysulfide complex apparently not available for methylation [28],[91].

In contrast, the analogous boundary separating predominance of Ag(HS)_2_^−^ from Ag(S_4_)_2_^3−^ lies at a_S_^0^ = 2.14 at pH 7 but falls to 0.96 at pH 8 (data from [23],[92]). Thus Ag(S_4_)_2_^3−^ is relatively less stable than Hg(S_n_)_2_^2−^ and likely to be important only at higher pH in the range of a_S_0 found at earth-surface temperatures in nature.

Similar calculations indicate that Sb^V^ thioanions will predominate over Sb^III^ thioanions [19], and As^V^ thioanions will predominate over As^III^ thioanions at a_S_0 values found in Table 1 and Figures 5 and 6[21]. Although in both cases improvements are needed in the thermodynamic data, these predictions of the importance of oxidized thioanions in reducing natural waters seem to be borne out qualitatively by field measurements [93]–[96].

Reaction of S^0^ with MoOS_3_^2−^ in sulfidic solution produces an unstable Mo(VI) intermediate, MoOS(S_2_)_2_^2−^, that undergoes spontaneous rearrangement to a Mo(IV) complex [20]. This may be the initiation step in the scavenging of Mo from sulfidic natural waters and might explain the reduced oxidation state of Mo in sediments [97]. From data in [20], the MoOS(S_2_)_2_^2−^/MoOS_3_^2−^ ratio would reach 0.1 at an a_S_0 value in the neighborhood of 0.5, a threshold exceeded in most of the sulfidic waters surveyed in this paper.

The a_S_0 values in Figures 5 and 6 and in Table 1 are quite high compared to equilibrium phase boundaries in several common mineral systems. The transformation of mackinawite to greigite,

15 $$3\mathrm{FeS}\;+\;S^{0}\to {\mathrm{Fe}}_{3}S_{4},$$

occurs at a_S_0 > 10^-2.91^ (taking log K_greig_ to be 2.24 from this work and log K_mack_ to be 3.21 [98]). Realgar becomes unstable relative to orpiment at a_S_0 > 10^-3.91^[21]. Chalcocite, djurleite and anilite all become unstable relative to covellite at a_S_0 > 10^-2.29^[25]. Thus the a_S_0 values in Figures 5 and 6 and Table 1 all imply that in each of these three mineral systems the most sulfur-rich mineral (greigite, orpiment, covellite)) would be the stable sulfide mineral.

## Conclusions

The activity of zero-valent sulfur is an important property of sulfidic waters, but one that cannot be measured directly. Methods for determining it from measurable properties of natural waters have been reviewed here. A critical test of the two principal methods against one another yields inconsistent results when ΣS^0^/ΣS^-II^ is low. The source of this problem appears to lie in the thermodynamic data, especially for disulfides. The inconsistencies largely can be removed by a pragmatic modification to accepted ionization constants for polysulfides, but this approach requires further investigation. A survey of a_S_0 in sulfidic natural waters yields values in the 0.3 to >1.2 range. Those values >1.2 are believed to be artifacts caused by inclusion of colloidal sulfur in ΣS^0^ determinations. In all of the surveyed environments, S^0^ is unstable with respect to SO_4_^2−^ and sulfide. In these environments, a_S_0 values are high enough to have a significant influence on the biogeochemical behavior of Cu, Hg, As, Sb, Mo and probably many other trace elements.

## Additional file

## Electronic supplementary material

Additional file 1: **With this paper is provided an additional file that consists of six parts.** **A)** Some additional details clarifying why equilibrium thermodynamic models are appropriate for describing zero-valent sulfur chemistry. **B)** Details concerning derivation of the equations associated with Method II. **C)** Thermodynamic data used in this paper. **D)** Full-page versions of Figures 1 to 3. **E)** Numerical tables relevant to the comparison of Methods I and II against one another. **F)** Tables illustrating propagation of analytical errors. (DOCX 436 KB)

Below are the links to the authors’ original submitted files for images.

Authors’ original file for figure 1

Authors’ original file for figure 2

Authors’ original file for figure 3

Authors’ original file for figure 4

Authors’ original file for figure 5

Authors’ original file for figure 6

Authors’ original file for figure 7
