# Supplementary material for: Activity of zero-valent sulfur in sulfidic natural waters
Source: Geochem Trans. 2014 Aug 19;15:13. doi: 10.1186/s12932-014-0013-x (PMC4631721; doi:10.1186/s12932-014-0013-x)
Supplement: Supplementary file 1 — Additional file 1: With this paper is provided an additional file that consists of six parts. A) Some additional details clarifying why equilibrium thermodynamic models are appropriate for describing zero-valent sulfur chemistry. B) Details concerning derivation of the equations associated with Method II. C) Thermodynamic data used in this paper. D) Full-page versions of Figures 1 to 3. E) Numerical tables relevant to the comparison of Methods I and II against one another. F) Tables illustrating propagation of analytical errors. (DOCX 436 KB) [file 12932_2014_13_MOESM1_ESM.docx]

**Additional file 1**

**Activity of zero-valent sulfur in sulfidic natural waters.**

George R. Helz

Chemistry and Biochemistry

University of Maryland

College Park, MD 20742

[helz@umd.edu](mailto:helz@umd.edu)

1. **Equilibrium in S^0^-containing systems.**

There is some confusion in the literature about whether polysulfide speciation in natural waters is controlled by thermodynamics. To clarify the issues, it is important first to distinguish between homogeneous and heterogeneous reactions. In homogeneous reactions, both reactants and products exist in the same phase, whereas in heterogeneous reactions they exist in different phases (aqueous vs. solid or aqueous vs. colloid). In the laboratory, Kamyshny et al. [1] showed that S-atom transfer among dissolved polysulfide ions is so fast that these ions come to homogeneous equilibrium in seconds. On the other hand, Wang and Tessier [2] (as well as others) showed that ~ 10 d is required for dissolved S_8_(aq) to come to heterogeneous equilibrium with rhombic sulfur (the rate will vary with particle size, stirring rate, pH and possibly other factors). Therefore, in natural waters that are subjected to relatively rapid chemical change (e.g. waters in tide pools, flowing ground water springs, tidal marshes) heterogeneous equilibrium would be difficult to maintain, but homogeneous equilibrium would be difficult to avoid. On the other hand, in waters that change very slowly (e.g. waters in aquifers, euxinic basins, deep sea sediment pore waters), both homogeneous and heterogeneous equilibrium must be expected.

Another important distinction concerns metastable vs. stable equilibrium. Rapid S-atom transfer between polysulfide ions keeps them close to equilibrium with one another, but this equilibrium is metastable, as demonstrated in Figure 7 of the main paper. Given sufficient time or given microbial catalysis, polysulfide ions will decompose to sulfide and sulfate; microbes are able to harvest energy by catalyzing this transformation [3]. An analogy can be drawn to CH_3_COOH and CH_3_COO^-^ (acetic acid/acetate) which equilibrate with one another very rapidly in aqueous solutions (thus acting as a superb pH buffer), but are always metastable with respect to CH_4_ and CO_2_.

In several papers, Boulègue has described certain natural water samples as being out of equilibrium with the H_2_S-S_8_-H_2_O system [4-6]. This diagnosis is rendered if the potentials of platinum and of Ag/Ag_2_S electrodes fail to change by the same amount when a sample is spiked with acid or base. In the terminology used in the present paper, this failure simply implies that a_S_0 in the sample is changing with pH and therefore is not fixed by saturation with respect to a condensed phase. Boulègue’s description, “out of equilibrium with the H_2_S-S_8_-H_2_O system,” characterizes a system that is undersaturated with a condensed sulfur phase, regardless of whether it is in homogeneous equilibrium.

Similarly, polysulfides in tide pools have been described as out of equilibrium because the observed ratio of different polysulfide ions is not equal to the ratio in equilibrium with rhombic sulfur or with colloidal sulfur, which was observed in the water [7]. This evidence excludes heterogeneous equilibrium but does not exclude existence of homogeneous equilibrium among the dissolved species.

1. **Derivation of equations employed in Method II.**

Following previous practice in which all H_x_S_n_^x-2^ polysulfides are imagined to consist of one S^-II^ and (n-1)S^0^ atoms, ΣS^0^ can be related to the molecular and ionic carriers of zero-valent sulfur by the following molar balance equation:

*ΣS^0^ = 8[S_8_](aq)+8[S_8_](sorb)] +7([S_8_^2-^] +[HS_8_^-^] + [H_2_S_8_^0^]) +*

*6([S_7_^2-^] +[HS_7_^-^] + [H_2_S_7_^0^]) + …([S_2_^2-^] +[HS_2_^-^] + [H_2_S_2_^0^])* (S1

This equation is simply a statement of what the analytical method for ΣS^0^ measures. It assumes that colloidal S is excluded from ΣS^0^, which is true of some, but not all analytical procedures [2,8,9].

The first term in equation 1 accounts for dissolved molecular S_8_. The second accounts for molecular S_8_ physically bound to filter-passing organic macromolecules (DOC). (It is assumed that the analytical procedure excludes zero-valent S bound to particulate organic carbon, POC, as well as to other kinds of particles.) The remaining terms (21 in all) account for S^0^ in all polysulfides containing 2 to 8 sulfur atoms and zero to two protons. Polysulfides with n > 8 exist [10] but are likely to be negligible at a_S_0 values attainable in natural waters. Equation 5 omits organic polysulfides and polysulfanes, as well as polythionates and higher valent thioanions like AsS_4_^3-^; these usually are negligible contributors to measured ΣS^0^ in natural waters [7-11].

By replacing the terms in equation 1 with mass action laws linking them to the master variables, pH, a_HS-_ and a_S_0_,_ the following equation is obtained.

$\Sigma S^{0}=8\left[ \frac{{(a_{S^{0}})^{8}K}_{0}}{\gamma_{0}}\left( 1+K_{p}(DOC) \right) \right]+\left( a_{HS^{-}} \right)\sum_{n=2}^{8} \left[ \frac{(n-1)(K_{n}a_{S^{0}}^{(n-1)})}{{10}^{-pH}}\left( \frac{1}{\gamma_{2}}+\frac{{10}^{-pH}}{K_{na2}\gamma_{1}}+\frac{{10}^{-2pH}}{K_{na1}K_{na2}\gamma_{0}} \right) \right]$ (S2

Where *γ_q_* represents the ionic activity coefficient of a species having charge *±q* (*γ_q_* is approximated in this work with the Davies equation). Based on data in [12], γ_o_, the activity coefficient of dissolved molecular S_8_ and all uncharged aqueous species, can be computed from a Setchenow relationship, γ_o_ = 10^0.31(^*^I^*^)^, where *I* is the ionic strength. Additional equilibrium constants in equation 2 are defined in the following section.

Unless ΣS^0^<<ΣS^-II^, evaluating a_HS-_ requires a correction for S^-II^ contained in polysulfides. This can be extracted from the following molar balance constraint:

*ΣS^-II^ = [H_2_S] + [HS^‒^] + [S_8_^2-^] + [HS_8_^‒^] + [H_2_S_8_^0^] + [S_7_^2-^] + [HS_7_^‒^] +…[S_2_^2-^] + [HS_2_^‒^] + [H_2_S_2_^0^]*  (S3

which assumes that labile sulfide complexes or colloids formed with metals like Fe are negligible [9]. Again replacing these concentration terms using equilibrium constant expressions yields:

$\Sigma S^{-II}=\left( a_{{HS}^{-}} \right)\left\{ \left( \frac{1}{\gamma_{1}}+\frac{{10}^{-pH}}{K_{a1}\gamma_{0}} \right)+\sum_{n=2}^{8} \left[ \frac{K_{n}a_{S^{0}}^{(n-1)}}{{10}^{-pH}}\left( \frac{1}{\gamma_{2}}+\frac{{10}^{-pH}}{K_{na2}\gamma_{1}}+\frac{{10}^{-2pH}}{K_{na1}K_{na2}\gamma_{0}} \right) \right] \right\}$ (S4

If dissolved free and DOC-sorbed molecular S_8_ (first term in brackets in equation 2) can be neglected, then dividing equation S2 by equation S4 yields the main paper’s equation 7, which is the basis for the main paper’s Figures 2 and 3.

**C. Thermodynamic data.**

Values for the equilibrium constants in equations S2 and S4 have been mostly obtained from Kamyshny and coworkers [12-14] and are summarized below (references in brackets).

8S^0^ ↔ S_8_(aq), $K_{0}=\frac{\gamma_{0}{[S}_{8}]}{(a_{S^{0}})^{8}}={10}^{\left( \frac{-2475}{T}+0.783 \right)}$ [12]

S_8_(aq) ↔ S_8_(sorb), $K_{p}= \frac{C_{S_{8}.DOC}}{{[S}_{8}]}\approx{10}^{5.1}$ for all T [9]

where C_S8,DOC_ represents moles S_8_ per kg of dissolved organic carbon and (DOC) in equation S2 is the concentration of dissolved organic carbon *expressed in kg C/L*. This value of K_p_ is based on one experiment with only a sinlge humic acid sample, but at present it is the best available value.

HS_n_^‒^ ↔ H^+^ + S_n_^2-^, $K_{na2}=\frac{{10}^{-pH}a_{S_{n}^{2-}}}{a_{HS_{n}^{-}}}$ = 10^-6.3^ for all n and all T. (see main text)

H_2_S_n_^0^ ↔ H^+^ + HS_n_^‒^, $K_{na1}=\frac{{10}^{-pH}a_{HS_{n}^{-}}}{a_{{H_{2}S}_{n}^{0}}}$ = 10^-3.0^ for all n and all T. (see main text)

H_2_S ↔ H^+^ + HS^-^ $K_{a1}=\frac{{10}^{-pH}a_{HS^{-}}}{a_{{H_{2}S}^{0}}}={10}^{-(-98.080+\frac{5765.4}{T}+15.0455lnT)}$ [15]

HS^-^ + (n-1)S^0^ ↔ H^+^ + S_n_^2-^, ${K_{n}= \frac{{{10}^{-pH}a}_{S_{n}^{2-}}}{a_{{HS}^{-}}(a_{S^{0}})^{(n-1)}}}$ Table below. [13]

| n | log K_n_ |
| --- | --- |
| 2 | -65.32/2.3R(298)+30.6*Δ(T^-1^)/2.3R |
| 3 | -59.02/2.3R(298)+24.2*Δ(T^-1^)/2.3R |
| 4 | -55.02/2.3R(298)+26.6*Δ(T^-1^)/2.3R |
| 5 | -53.92/2.3R(298)+27.2*Δ(T^-1^)/2.3R |
| 6 | -55.32/2.3R(298)+30.9*Δ(T^-1^)/2.3R |
| 7 | -58.62/2.3R(298)+34.1*Δ(T^-1^)/2.3R |
| 8 | -62.82/2.3R(298)+41.1*Δ(T^-1^)/2.3R |

Where:

T = temperature (K)

R = Gas constant, 0.00831 kJ/mol/^o^K

$$\Delta\left( T^{-1} \right)=\left( \frac{1}{298}-\frac{1}{T} \right)$$

**D. Larger versions of Figures 1 to 3 in the main paper (for graphically solving for a_S_0).**

**E. Tests of Methods I vs. II in the waters of wells Kfar Urya 8 and Tzofar 221.**

Table S-2 and S-3 present a comparison of computational results by Methods I and II for the waters of wells Kfar Urya 8 and Tzofar 221 described by Kamyshny et al. [16]. That study is extraordinary in that extensively redundant analytical data were acquired. Of the nine wells discussed, I chose two for which the alternate ΣS^0^ determinations by chloroform extraction and cyanolysis were reasonably consistent. Importantly, well Kfar Urya 8 had a ~25-fold higher ΣS^0^/ΣS^-II^ ratio than well Tzofar 221.

According to Method I, concentrations of two polysulfide ions define both a_S_0 and a_HS-_ in a sample, and from these two quantities, the equilibrium concentrations of all H_x_S_n_^x-2^ species as well as S_8_(aq), ΣS^0^ and ΣS^-II^ can be calculated, given pH, ionic strength and temperature. Method II yields the concentrations of all species from input values of ΣS^0^ and ΣS^-II^ (as well as pH, ionic strength and temperature). Tables S-2 and S-3 present redundant chemical speciation calculations obtained with different choices of input data. In each column, the two input values are shown in bold type and the resulting output values are shown in normal font. For both tables, section A is based on calculations using Kamyshny’s [13,14] thermodynamic data, whereas section B is based on partially suppressing protonated species by assigning pK_a2_ values of 6.3 and pK_a1_ values of 3.0 for all n in H_x_S_n_^x-2^. (The pK_a1_ values are irrelevant at the pH of these samples.) Reasons for this modification to the data are discussed in the main paper. Figure 4 in the main paper is a graphical presentation of the numbers in Table S-3B.

**F. Propagation of analytical errors.**

Table S-4 presents median logarithms ± one half the logarithmic range of molar concentrations of polysulfides in well **Tzofar 221** [16]. These values are given in logarithmic format because in some cases analytical error is producing order of magnitude uncertainties in calculated values. Bold type indicates logarithms of the input analytical data with uncertainty as estimated by the original authors [16]; normal type indicates values calculated from the same thermodynamic model used for Table S-3B (i.e. all pK_a2_ values set to 6.3 and all pK_a1_ values set to 3.0).

To illustrate how the numbers in this table were derived, consider the first case, in which species were calculated from the S_7_^2-^ and S_6_^2-^ data obtained by the methyl triflate derivatization method. From [16], the molar concentration of S_7_^2-^ was 0.27±0.13 μM and the molar concentration of S_6_^2-^ was 0.50±0.14 μM. Two species calculations were done with these data. In the first, the maximum value of S_7_^2-^ (0.27+0.13 μM) was combined with the minimum value of S_6_^2-^ (0.50‒0.14 μM). In the second, the minimum value of S_7_^2-^ (0.27‒0.13 μM) was combined with the maximum value of S_6_^2-^ (0.50+0.14 μM). The midpoint of the logarithms of all concentrations obtained in these two calculations is reported in the table with an uncertainty of ±½ the range in these logarithms. In the case of Method II, minimum and maximum values of ΣS^0^ and ΣS^-II^ were treated in the same way.

*Table S-2. Comparison of redundant species calculations by Methods I and II using different input data from well* ***Kfar Urya 8;*** *In part A, the themodynamic data of Kamyshny [13,14] were used as presented. Calculated values are in normal font; analytical input for calculations in bold font. In part B, all pK_a2_ values for polysulfides were set to 6.3 and pK_a1_ were set to 3.0. In all calculations,* I = 0.028 M; T = 29^o^C; pH = 7.33.

| **A.** | Method I  S_6_^2-^/S_5_^2-^ | | Method I  S_5_^2-^/S_4_^2-^ | Method I  S_6_^2-^/S_4_^2-^ |  | Method II  chloroform | Method II  cyanolysis | Method II  Σ(n-1)(S_4_ to S_7_)* |
| --- | --- | --- | --- | --- | --- | --- | --- | --- |
| ΣS^-II^ (μM) | 9.6 | 60. | | 30. |  | **27** | **27** | **27** |
| ΣS^0^ (μM) | 4.1 | 3.7 | | 3.9 |  | **5.5** | **5.0** | **2.0** |
| a_S_0 | 1.21 | 0.75 | | 0.95 |  | 1.07 | 1.05 | 0.80 |
| HS^-^ (μM) | 6.5 | 43 | | 21 |  | 19 | 19 | 19 |
| S_8_^0^(aq) (μM) | 0.17 | 0.004 | | 0.026 |  | 0.068 | 0.055 | 0.006 |
| S_8_^2-^ (μM) | 0.010 | 0.003 | | 0.007 |  | 0.013 | 0.011 | 0.002 |
| S_7_^2-^ (μM) | 0.045 | 0.018 | | 0.036 |  | 0.064 | 0.056 | 0.011 |
| S_6_^2-^ (μM) | **0.14** | 0.087 | | **0.14** |  | 0.22 | 0.20 | 0.052 |
| S_5_^2-^ (μM) | **0.20** | **0.20** | | 0.253 |  | 0.36 | 0.33 | 0.11 |
| S_4_^2-^ (μM) | 0.11 | **0.17** | | **0.17** |  | 0.21 | 0.20 | 0.091 |
| S_3_^2-^ (μM) | 0.017 | 0.044 | | 0.035 |  | 0.039 | 0.038 | 0.022 |
| S_2_^2-^ (μM) | 0.001 | 0.005 | | 0.003 |  | 0.003 | 0.003 | 0.002 |
| HS_6_^-^ (μM) | 0.001 | 0.001 | | 0.001 |  | 0.002 | 0.002 | 0.000 |
| HS_5_^-^ (μM) | 0.006 | 0.006 | | 0.008 |  | 0.011 | 0.010 | 0.003 |
| HS_4_^-^ (μM) | 0.013 | 0.021 | | 0.021 |  | 0.026 | 0.024 | 0.011 |
| HS_3_^-^ (μM) | 0.033 | 0.086 | | 0.068 |  | 0.076 | 0.073 | 0.043 |
| HS_2_^-^ (μM) | 0.36 | 1.48 | | 0.92 |  | 0.92 | 0.90 | 0.71 |
| H_2_S_2_^0^ (μM) | 0.002 | 0.007 | | 0.007 |  | 0.005 | 0.005 | 0.004 |

* Sum of S^0^ contained in S_4_^2-^ to S_7_^2-^ as measured by triflate method; this is a minimum estimate of ΣS^0^.

| **B.** | Method I  S_6_^2-^/S_5_^2-^ | | Method I  S_5_^2-^/S_4_^2-^ | Method I  S_6_^2-^/S_4_^2-^ |  | Method II  chloroform | Method II  cyanolysis | Method II  Σ(n-1)(S_4_ to S_7_) |
| --- | --- | --- | --- | --- | --- | --- | --- | --- |
| ΣS^-II^ (μM) | 9.2 | 58 | | 29 |  | **27** | **27** | **27** |
| ΣS^0^ (μM) | 3.7 | 2.1 | | 2.9 |  | **5.5** | **5.0** | **2.0** |
| a_S_0 | 1.21 | 0.75 | | 0.95 |  | 1.11 | 1.09 | 0.89 |
| HS^-^ (μM) | 6.5 | 43.1 | | 21.2 |  | 19.5 | 19.5 | 20.0 |
| S_8_^0^(aq) (μM) | 0.173 | 0.004 | | 0.026 |  | 0.088 | 0.075 | 0.015 |
| S_8_^2-^ (μM) | 0.010 | 0.003 | | 0.007 |  | 0.017 | 0.015 | 0.004 |
| S_7_^2-^ (μM) | 0.045 | 0.018 | | 0.036 |  | 0.082 | 0.073 | 0.022 |
| S_6_^2-^ (μM) | **0.14** | 0.087 | | **0.14** |  | 0.28 | 0.25 | 0.092 |
| S_5_^2-^ (μM) | **0.20** | **0.20** | | 0.25 |  | 0.43 | 0.40 | 0.18 |
| S_4_^2-^ (μM) | 0.106 | **0.17** | | **0.17** |  | 0.25 | 0.23 | 0.13 |
| S_3_^2-^ (μM) | 0.017 | 0.044 | | 0.035 |  | 0.044 | 0.042 | 0.029 |
| S_2_^2-^ (μM) | 0.001 | 0.005 | | 0.003 |  | 0.003 | 0.003 | 0.003 |
| HS_6_^-^ (μM) | 0.008 | 0.005 | | 0.008 |  | 0.016 | 0.014 | 0.005 |
| HS_5_^-^ (μM) | 0.011 | 0.011 | | 0.014 |  | 0.024 | 0.023 | 0.010 |
| HS_4_^-^ (μM) | 0.006 | 0.010 | | 0.010 |  | 0.014 | 0.013 | 0.007 |
| HS_3_^-^ (μM) | 0.001 | 0.003 | | 0.002 |  | 0.002 | 0.002 | 0.002 |
| HS_2_^-^ (μM) | <0.001 | <0.001 | | <0.001 |  | <0.001 | <0.001 | <0.001 |
| H_2_S_2_^0^ (μM) | <0.001 | <0.001 | | <0.001 |  | <0.001 | <0.001 | <0.001 |

*Table S-3. Comparison of redundant species calculations by Methods I and II using different input data from well* ***Tzofar 221.*** *In part A, the themodynamic data of Kamyshny [13,14] were used as presented. Calculated values are in normal font; analytical input for calculations in bold font. In part B, all pK_a2_ for polysulfides were set to 6.3 and pK_a1_ were set to 3.0. In all calculations,* I = 0.041 M; T = 38^o^C; pH = 6.81.

| **A.** | Method I  S_7_^2-^/S_6_^2-^ | Method I  S_6_^2-^/S_5_^2-^ | | Method I  S_5_^2-^/S_4_^2-^ | Method I  S_6_^2-^/S_4_^2-^ |  | Method II  chloroform | Method II  cyanolysis | Method II  Σ(n-1)(S_4_ to S_7_) |
| --- | --- | --- | --- | --- | --- | --- | --- | --- | --- |
| ΣS^-II^ (μM) | 11 | 370 | 3400 | | 1460 |  | **770** | **770** | **770** |
| ΣS^0^ (μM) | 112 | 24 | 79 | | 53 |  | **5.3** | **4.0** | **11** |
| a_S_0 | 1.94 | 0.92 | 0.52 | | 0.69 |  | 0.18 | 0.13 | 0.36 |
| HS^-^ (μM) | 4.6 | 190 | 1800 | | 785 |  | 430 | 420 | 420 |
| S_8_^0^(aq) (μM) | 13 | 0.033 | 0.0004 | | 0.004 |  | <0.001 | <0.001 | <0.001 |
| S_8_^2-^ (μM) | 0.11 | 0.024 | 0.005 | | 0.014 |  | <0.001 | <0.001 | <0.001 |
| S_7_^2-^ (μM) | **0.27** | 0.13 | 0.042 | | 0.097 |  | <0.001 | <0.001 | 0.001 |
| S_6_^2-^ (μM) | **0.50** | **0.50** | 0.29 | | **0.50** |  | <0.001 | <0.001 | 0.009 |
| S_5_^2-^ (μM) | 0.43 | **0.90** | **0.90** | | 1.2 |  | 0.003 | 0.001 | 0.044 |
| S_4_^2-^ (μM) | 0.14 | 0.62 | **1.09** | | **1.09** |  | 0.011 | 0.004 | 0.079 |
| S_3_^2-^ (μM) | 0.014 | 0.13 | 0.40 | | 0.30 |  | 0.011 | 0.007 | 0.042 |
| S_2_^2-^ (μM) | 0.001 | 0.012 | 0.067 | | 0.038 |  | 0.005 | 0.004 | 0.010 |
| HS_6_^-^ (μM) | 0.014 | 0.014 | 0.008 | | 0.014 |  | <0.001 | <0.001 | <0.001 |
| HS_5_^-^ (μM) | 0.040 | 0.084 | 0.084 | | 0.11 |  | <0.001 | <0.001 | 0.004 |
| HS_4_^-^ (μM) | 0.052 | 0.23 | 0.40 | | 0.40 |  | 0.004 | 0.002 | 0.029 |
| HS_3_^-^ (μM) | 0.081 | 0.76 | 2.3 | | 1.762 |  | 0.066 | 0.040 | 0.248 |
| HS_2_^-^ (μM) | 0.58 | 12 | 62 | | 35 |  | 5.0 | 3.8 | 9.7 |
| H_2_S_2_^0^ (μM) | 0.009 | 0.18 | 0.99 | | 0.56 |  | 0.080 | 0.061 | 0.16 |

| **B.** | Method I  S_7_^2-^/S_6_^2-^ | Method I  S_6_^2-^/S_5_^2-^ | | Method I  S_5_^2-^/S_4_^2-^ | Method I  S_6_^2-^/S_4_^2-^ |  | Method II  chloroform | Method II  cyanolysis | Method II  Σ(n-1)(S_4_ to S_7_) |
| --- | --- | --- | --- | --- | --- | --- | --- | --- | --- |
| ΣS^-II^ (μM) | 10.1 | 353 | 3300 | | 1420 |  | **770** | **770** | **770** |
| ΣS^0^ (μM) | 112 | 11.0 | 11.1 | | 13.9 |  | **5.3** | **4.0** | **11** |
| a_S_0 | 1.94 | 0.92 | 0.52 | | 0.69 |  | 0.63 | 0.59 | 0.76 |
| HS^-^ (μM) | 4.6 | 194 | 1820 | | 785 |  | 425 | 425 | 424 |
| S_8_^0^(aq) (μM) | 12.9 | 0.033 | <0.001 | | 0.004 |  | 0.002 | 0.001 | 0.007 |
| S_8_^2-^ (μM) | 0.109 | 0.024 | 0.005 | | 0.014 |  | 0.004 | 0.002 | 0.015 |
| S_7_^2-^ (μM) | **0.27** | 0.128 | 0.042 | | 0.097 |  | 0.030 | 0.020 | 0.092 |
| S_6_^2-^ (μM) | **0.50** | **0.50** | 0.286 | | **0.500** |  | 0.172 | 0.119 | 0.433 |
| S_5_^2-^ (μM) | 0.427 | **0.90** | **0.90** | | 1.19 |  | 0.449 | 0.335 | 0.938 |
| S_4_^2-^ (μM) | 0.140 | 0.623 | **1.09** | | **1.09** |  | 0.450 | 0.361 | 0.781 |
| S_3_^2-^ (μM) | 0.014 | 0.130 | 0.398 | | 0.301 |  | 0.136 | 0.117 | 0.196 |
| S_2_^2-^ (μM) | 0.001 | 0.012 | 0.067 | | 0.038 |  | 0.019 | 0.017 | 0.023 |
| HS_6_^-^ (μM) | 0.087 | 0.087 | 0.049 | | 0.087 |  | 0.030 | 0.021 | 0.075 |
| HS_5_^-^ (μM) | 0.074 | 0.156 | 0.156 | | 0.206 |  | 0.078 | 0.058 | 0.162 |
| HS_4_^-^ (μM) | 0.024 | 0.108 | 0.189 | | 0.189 |  | 0.078 | 0.062 | 0.135 |
| HS_3_^-^ (μM) | 0.002 | 0.022 | 0.069 | | 0.052 |  | 0.023 | 0.020 | 0.034 |
| HS_2_^-^ (μM) | <0.001 | 0.002 | 0.012 | | 0.007 |  | 0.003 | 0.003 | 0.004 |
| H_2_S_2_^0^ (μM) | <0.001 | <0.001 | <0.001 | | <0.001 |  | <0.001 | <0.001 | <0.001 |

*Table S-4. Sensitivity of computed species concentrations to analytical error. This table shows five species calculations for water from the* ***Tzofar 221*** *well [16]. The first four are by Method I and differ according to which pair of polysulfide ion concentrations were used as input data. Logarithms of input data and estimated analytical uncertainty are given in bold type for each calculation. The fifth calculation was by Method II, making use of ΣS^-II^ determined by the Cline method and ΣS^0^ determined by the chloroform extraction method (see [16] for analytical methods). Values shown are midpoint log_10_(C) values ± ½ the range, where C is molar concentration.*

| Method🡪 | S_7_^2-^/ | S_6_^2-^ | S_6_^2-^/ | S_5_^2-^ | S_5_^2-^/ | S_4_^2-^ | S_6_^2-^/ | S_4_^2-^ | Chloro. | Extraction |
| --- | --- | --- | --- | --- | --- | --- | --- | --- | --- | --- |
| log ΣS^-II^ | -4.43 | ±1.37 | -3.57 | ±0.95 | -2.58 | ±0.80 | -3.00 | ±0.56 | **-3.11** | **±0.02** |
| log ΣS^0^ | -3.04 | ±1.74 | -4.65 | ±0.24 | -4.91 | ±0.02 | -4.84 | ±0.01 | **-5.28** | **±0.03** |
| log a_S_0 | 0.27 | ±0.35 | 0.00 | ±0.22 | -0.25 | ±0.22 | -0.12 | ±0.14 | -0.19 | ±0.01 |
| log HS^-^ | -5.21 | ±1.89 | -3.85 | ±0.97 | -2.84 | ±0.80 | -3.27 | ±0.56 | -3.38 | ±0.02 |
| log S_8_^0^ | -5.02 | ±2.82 | -7.19 | ±1.74 | -9.21 | ±1.80 | -8.13 | ±1.10 | -8.73 | ±0.11 |
| log S_8_^2-^ | -7.11 | ±0.58 | -7.66 | ±0.56 | -8.41 | ±0.77 | -7.89 | ±0.40 | -8.53 | ±0.08 |
| log S_7_^2-^ | **-6.62** | **±0.23** | -6.90 | ±0.34 | -7.40 | ±0.54 | -7.01 | ±0.26 | -7.58 | ±0.06 |
| log S_6_^2-^ | **-6.32** | **±0.12** | **-6.32** | **±0.12** | -6.57 | ±0.32 | **-6.32** | **±0.12** | -6.80 | ±0.05 |
| log S_5_^2-^ | -6.33 | ±0.48 | **-6.06** | **±0.09** | **-6.06** | **±0.09** | -5.94 | ±0.01 | -6.35 | ±0.04 |
| log S_4_^2-^ | -6.78 | ±0.83 | -6.23 | ±0.31 | **-5.98** | **±0.13** | **-5.98** | **±0.13** | -6.34 | ±0.02 |
| log S_3_^2-^ | -7.70 | ±1.18 | -6.89 | ±0.53 | -6.38 | ±0.36 | -6.54 | ±0.29 | -6.80 | ±0.01 |
| log S_2_^2-^ | -9.08 | ±1.54 | -7.99 | ±0.75 | -7.23 | ±0.58 | -7.52 | ±0.42 | -7.71 | ±0.01 |
| log HS_6_^-^ | -7.07 | ±0.12 | -7.07 | ±0.12 | -7.32 | ±0.32 | -7.07 | ±0.12 | -7.56 | ±0.05 |
| log HS_5_^-^ | -7.08 | ±0.48 | -6.81 | ±0.09 | -6.81 | ±0.09 | -6.69 | ±0.01 | -7.10 | ±0.04 |
| log HS_4_^-^ | -7.53 | ±0.83 | -6.99 | ±0.31 | -6.74 | ±0.13 | -6.75 | ±0.15 | -7.09 | ±0.02 |
| log HS_3_^-^ | -8.45 | ±1.18 | -7.64 | ±0.53 | -7.14 | ±0.36 | -7.29 | ±0.29 | -7.55 | ±0.01 |
| log HS_2_^-^ | -9.83 | ±1.54 | -8.74 | ±0.75 | -7.99 | ±0.58 | -8.27 | ±0.42 | -8.46 | ±0.01 |
| log H_2_S_2_^0^ | -13.73 | ±1.54 | -12.65 | ±0.75 | -11.89 | ±0.58 | -12.18 | ±0.42 | -12.36 | ±0.01 |

**References**

1. Kamyshny A. Jr., Goifman, A., Rozkov D., Lev O.: Kinetics of disproportionation of inorganic polysulfides in undersaturated aqueous solutions at environmentally relevant conditions. *Aqatic Geochem*. 2003, 9:291-304.

1. Wang F., Tessier A.: Zero-valent sulfur and metal speciation in sediment porewaters of freshwater lakes. *Environ. Sci. Technol*. 2009, 43:7252-7257.
2. Thamdrup B., Finster K., Hansen J. W., Bak F.: Bacterial disproportionation of elemental sulfur coupled to chemical reduction of iron or manganese. *Appl. Environ. Microbiol.* 1993, 59:101-108.
3. Boulegue J.: Equilibria in a sulfide rich water from Enghien-les-Bains, France. *Geochim. Cosmochim. Acta*, 1977, 41:1751-1758.
4. Boulegue J., Michard G.: Sulfur speciations and redox processes in reducing environments. *In Chemical Modeling in Aqueous Systems,* Edited by Jenne E. A. American Chemical Society Symposium Series 93, 1979: 25-50.
5. Boulegue J., Lord S. J. III, Church T. M.: Sulfur speciation and associated trace metals (Fe,Cu) in the pore waters of Great Marsh, Delaware. *Geochim. Cosmochim. Acta*, 1982, 46:453-464.
6. Kamyshny A. Jr., Ferdelman T. G.: Dynamics of zero-valent sulfur species including polysulfides at seep sites on intertidal sand flats (Wadden Sea, North Sea). *Mar. Chem*., 2010, 121:17-26.
7. Kamyshny A. Jr., Borkenstein C. G., Ferdelman T. G.: Protocol for quantitative detection of elemental sulfur and polysulfide zero-valent sulfur distribution in natural aquatic samples.  *Geostand. Geoanal. Res*. 2009, 33:415-435.
8. Bura-Nakić E., Helz G. R., Ciglenečki I., Ćosović B.: Reduced sulfur species in a stratified seawater lake (Rogoznica Lake, Croatia); seasonal variations and argument for organic carriers of reactive sulfur. *Geochim. Cosmochim. Acta*, 2009, 73:3738-3751.
9. Gun J, Modestov A. D., Kamyshny A., Ryzkov D., Gitis V., Goifman A., Lev O., Hultsch V., Grischek T, Worch E.: Electrospray ionization mass spectrometric analysis of aqueous polysulfide solutions. *Microchim. Acta,* 2004, 146:229-237.
10. Zopfi J., Ferdelman T. G., Fossing H.: Distribution and fate of sulfur intermediates—sulfite, tetrathionate, thiosulfate and elemental sulfur—in marine sediments. *Geological Society of American Special Paper 379*, 2004: 97-116.
11. Kamyshny A. Jr.: Solubility of cyclooctasulfur in pure water and sea water at different temperatures. *Geochim. Cosmochim. Acta*, 2009, 73:6022-6028.
12. Kamyshny A. Jr., Gun J., Rozkov D., Voitsekovski T., Lev O.: Equilibrium distribution of polysulfide ions in aqueous solutions at different temperatures by rapid single phase derivatization. *Environ. Sci. Technol*. 2007, 41:2395-2400.
13. Kamyshny A. Jr., Goifman A., Gun J., Rizkov D., Lev O.: Equilibrium distribution of polysulfide ions in aqueous solutions at 25^o^C: A new approach for the study of polysulfides’ equilibria. *Environ. Sci. Technol*. 2004, 38:6633-6644.
14. Millero F. J., Plese T., Fernandez M.: The dissociation of hydrogen sulfide in seawater. *Limnol. Oceanogr.* 1988, 33:2269-274.
15. Kamyshny A. Jr., Zilerbrand M., Ekeltchik I., Voitsekovski T., Gun J., Lev O.: Speciation of polysulfides and zerovalent sulfur in sulfide-rich water wells in southern and central Israel. *Aquat. Geochem.* 2008, 14:171-192.
