# Supplementary figures and images for: Activity of zero-valent sulfur in sulfidic natural waters
Source: Geochem Trans. 2014 Aug 19;15:13. doi: 10.1186/s12932-014-0013-x (PMC4631721; doi:10.1186/s12932-014-0013-x)

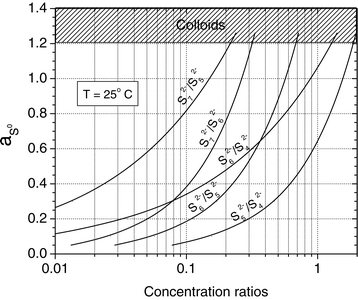

Supplement: Supplementary file 2 — Authors’ original file for figure 1 [file 12932_2014_13_MOESM2_ESM.gif]

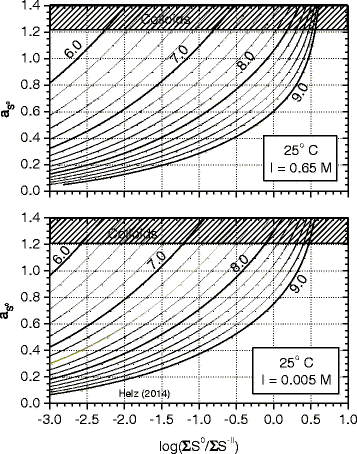

Supplement: Supplementary file 3 — Authors’ original file for figure 2 [file 12932_2014_13_MOESM3_ESM.gif]

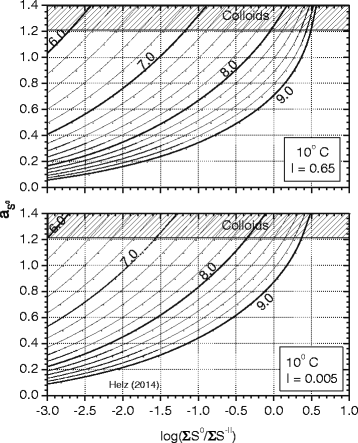

Supplement: Supplementary file 4 — Authors’ original file for figure 3 [file 12932_2014_13_MOESM4_ESM.gif]

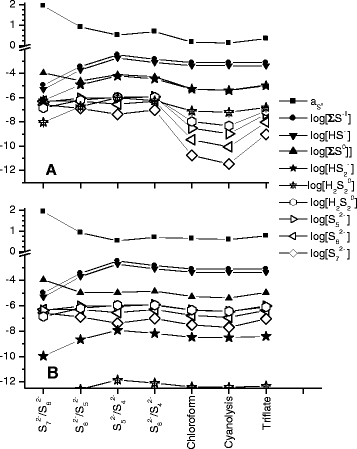

Supplement: Supplementary file 5 — Authors’ original file for figure 4 [file 12932_2014_13_MOESM5_ESM.gif]

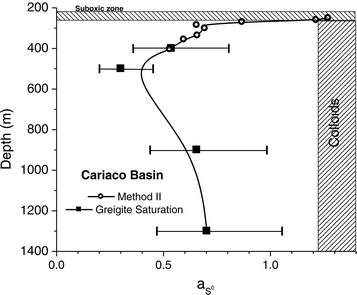

Supplement: Supplementary file 6 — Authors’ original file for figure 5 [file 12932_2014_13_MOESM6_ESM.gif]

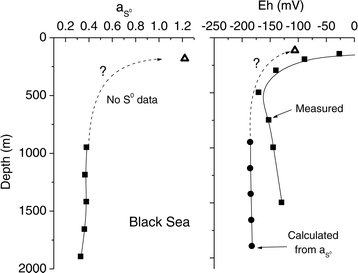

Supplement: Supplementary file 7 — Authors’ original file for figure 6 [file 12932_2014_13_MOESM7_ESM.gif]

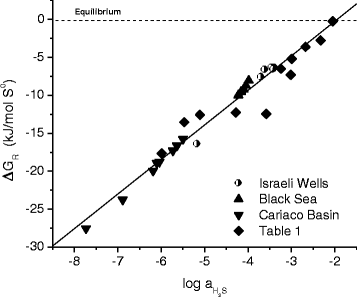

Supplement: Supplementary file 8 — Authors’ original file for figure 7 [file 12932_2014_13_MOESM8_ESM.gif]
